# Rare genetic disease in China: a call to improve clinical services

**DOI:** 10.1186/s13023-015-0333-7

**Published:** 2015-10-29

**Authors:** M. Chopra, T. Duan

**Affiliations:** Shanghai First Maternity and Infant Hospital, Tongji University School of Medicine, Shanghai, China; Department of Medical Genomics, Royal Prince Alfred Hospital, Missenden Road, Camperdown, Sydney, Australia; University of Sydney, School of Genetic Medicine and Centre for China Studies, Sydney, Australia

Patients with rare genetic diseases are becoming increasingly recognised worldwide as an important public health challenge. Despite dramatic advances in public medicine and genomic research in China, the needs of the rare disease patient population are far from being met.

Lack of accurate epidemiological data in China means that the true number of patients affected with a rare disease is unknown. 10 million is often cited, less than 1 % of the nation’s population of 1.3 billion [1, 2]. This is likely to be a gross underestimate. Rare diseases, most of them being genetic in origin, are often chronic, disabling and/or life—limiting [1]. Accepting that rare genetic disease is an enormous public health challenge is the first step to health care and policy reform to better serve the needs of this patient population, especially now that science is generating results which can directly benefit this population with enormous unmet needs. A rigorous epidemiological study is needed to understand the extent of this challenge in the world’s most populous nation.

A number of important rare disease initiatives in China have been previously reported, including the establishment of patient registries, increased funding for translational research and advocacy for orphan drug legislation [1–3]. While there is no doubt that a response to rare disease in China needs to be multifaceted—as it has been in Western countries—we believe that the most important priority at present should be to systematically improve the *quality* and *accessibility* of services for the diagnosis of rare genetic disease.

Clinical Genetics services are offered in general hospitals in China mainly through obstetrics and paediatrics [4], but there is no minimum standard of care for the provision of such services. The Ministry of Health does not recognise Clinical Genetics as a medical specialty and no formal training program exists to qualify those obstetricians, paediatricians and laboratory personnel who practice in this field. These physicians are under enormous pressure to see patients quickly, with limited time to perform the detailed clinical assessments and extensive literature searching that rare disease patients so often require. Accessing these services is a further challenge. With 50 % of patients in China living in rural centres [5], distance, cost and a fragmented province—based health system serve as barriers to access, as services are concentrated in the most populous cities. In non-metropolitan areas, there is lack of awareness amongst health professionals about the referral pathway for patients with undiagnosed genetic conditions [6].

While next generation sequencing rapidly progresses in China, a number of barriers limit the utilisation of this technology for the benefit of patients with rare genetic disease. Top-tier universities and tertiary hospitals offer molecular genetic testing on a research basis but laboratory reports are rarely issued [5]. This means that results are seldom able to be used for management or reproductive choices. Conversely, commercial genomics laboratories offer next generation sequencing “diagnostic” testing at a cost. While 90 % of China’s population are covered under a universal insurance scheme, genetic testing is excluded from coverage, so the cost is prohibitive to many patients [6]. The biggest barrier is the discrepancy between ability to genotype and skills to phenotype. While it is now relatively easy to generate vast quantities of genomic data, skills for phenotyping—essential for the interpretation of genetic tests and in predicting clinical course and mortality in rare disease [6, 7] —are lacking.

Physicians and laboratories are challenged by the complex regulatory guidelines around genetic testing. As recently as February 2014, genetic testing was banned by the Chinese FDA [8]. The wording of the ban was ambiguous, leaving physicians and laboratories doubtful about whether only non-invasive prenatal genomic sequencing or all genetic testing was covered by the ban. The ban has since been lifted, but physicians remain uneasy about the legal framework around using results of genetic testing for decision-making, particularly in the prenatal setting. The Chinese FDA requires that every reagent and sequencing machine in a given laboratory is licensed for genetic testing rather than the laboratory itself. Many laboratories operate outside of these cumbersome legal requirements, a factor which contributes to the reluctance to issue diagnostic reports.

For rare disease patients unable to access a high quality diagnostic assessment and pro-active management services, the medical, social and economic consequences have been well –documented [9]. Patients may receive erroneous diagnoses and inappropriate or inadequate management [9, 10]. Without an accurate molecular diagnosis, genetic counselling and where appropriate, prenatal genetic testing for family members at risk of recurrence cannot be offered.

There have been dramatic improvements in health care in China in recent decades, reflected by rapid improvements in life expectancy and infant mortality rates, which are now close to those achieved in developed countries [11, 12]. Medical care for patients with rare genetic disease lags far behind. This public health challenge needs to be addressed by improving the level of training in genetic disease amongst all health professionals, launching a formal professional qualification in Clinical Genetics and establishing local and national referral centres for clinical assessment and management of rare genetic disease. The regulations around genetic testing need to be simplified and a nationally recognised accreditation scheme for molecular genetic laboratories—be they public or commercial –needs to be introduced. Efforts such as establishing patient registries and support for orphan drug legislation must continue but their value can only be realised if rare disease patients have access to quality diagnostic services.

Note: Both authors contributed to development of ideas and in writing of this paper. There are no conflicts of interest. There are no funding declarations.
